# Allosteric Mechanisms Triggering Substrate and Cofactor Binding in the SULT1A1 Dimer as Revealed by Molecular Dynamics Simulations

**DOI:** 10.1021/acs.jcim.5c00845

**Published:** 2025-09-30

**Authors:** Daniel Toth, Balint Dudas, Arnaud B. Nicot, Maria A. Miteva, Erika Balog

**Affiliations:** † Department of Biophysics and Radiation Biology, Semmelweis University, 1094 Budapest, Hungary; ‡ Université Paris Cité, CiTCoM UMR 8038 CNRS, INSERM U1268 MCTR, 75006 Paris, France; § Laboratory of Computational Biology, National Heart, Lung, and Blood Institute, National Institutes of Health, Bethesda, Maryland 20892, United States; ∥ INSERM UMR 1064, Nantes Université, CR2TI, 44000 Nantes, France

## Abstract

Sulfotransferases (SULTs) are phase II drug-metabolizing enzymes metabolizing a wide range of endogenous compounds and xenobiotics including drugs. SULTs form dimers in vivo, and most isoforms share a conserved dimerization motif. Since it has been shown that the monomers of the SULT1A1 isoform maintain their activity in vitro, the biological significance of dimerization remains unclear. To elucidate the mechanism and the effects of dimerization on the SULT1A1 structure and function, we performed molecular dynamics (MD) simulations on both the monomer and dimer form of the enzyme and investigated the effect of cofactor and substrate binding into the dimer structure and dynamics. Our results show a clear dynamical effect on the dimerization of the apoenzyme, resulting in an increase of the ligand binding gate opening and greater fluctuation of the functional loops of one monomeric subunit. Furthermore, in the dimer, we uncovered intra- and intersubunit allosteric effects as a direct consequence of cofactor and the substrate binding, and we present the corresponding allosteric pathways. Our analyses suggest that the asymmetric behavior of the dimer in the presence of one PAPS molecule may reflect a half-site reactivity mechanism, previously suggested for SULT dimer function, which may be particularly important for large substrates. Thus, our study shed new light in our understanding of SULT1A1 structural dynamics and dimerization as related to enzyme function.

## Graphical abstract

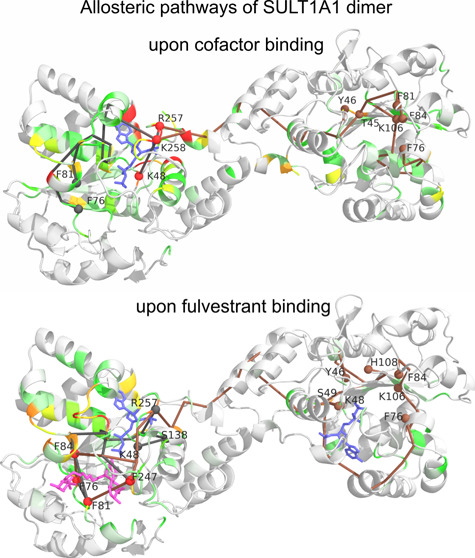

## 1 Introduction

Sulfotransferases (SULTs) are drug-metabolizing enzymes (DMEs) that catalyze an important conjugation reaction in phase II drug metabolism. The sulfonate (SO_3_^–^) transfer from the SULT cofactor, 3′-phosphoadenosine 5′-phosphosulfate (PAPS), on many xenobiotics and endogenous small molecules facilitates the elimination of the sulfated, water-soluble products.^1,2^ Diminished SULT activity due to genotype variants^3^ or drug–drug interactions (DDIs) could lead to accumulation of toxic compounds provoking adverse drug reactions, the fourth leading cause of death worldwide.^4^

SULTs, like other major DMEs,^5,6^ exhibit a broad range of substrates and display important structural plasticity, which has been extensively studied in recent decades.^1,7,8^ Their promiscuity is the result of their structural composition, which comprises a rigid core and an active site surrounded by flexible loops. As seen in Figure 1A, the core of SULT1A1 is composed of a central four-stranded parallel β-sheet surrounded by α-helices. The active site where the catalytic reaction takes place includes the substrate and PAPS binding pockets. Three flexible loops cover the active site, shown as L1, L2, and L3. L1, which is commonly termed Lip (residues 83–91), is characterized by high flexibility, modulating the access of substrates to the ligand binding residues within the pocket. In contrast, L2 (residues 141–158) was found to display minimal flexibility.^9^ However, it contains several crucial residues that are essential for substrate binding and the catalytic function of the enzyme.^10^ The largest loop can be subdivided into three distinct regions: L3 (which is also known as Cap) defined by residues 241–255, containing the substrate binding amino acids;^1,7^ the region over the PAPS binding pocket (residues 256–262), named here cL3, and the N-terminal side of L3 consisting of residues 236–240 named nL3 in this article.

**Figure 1:**
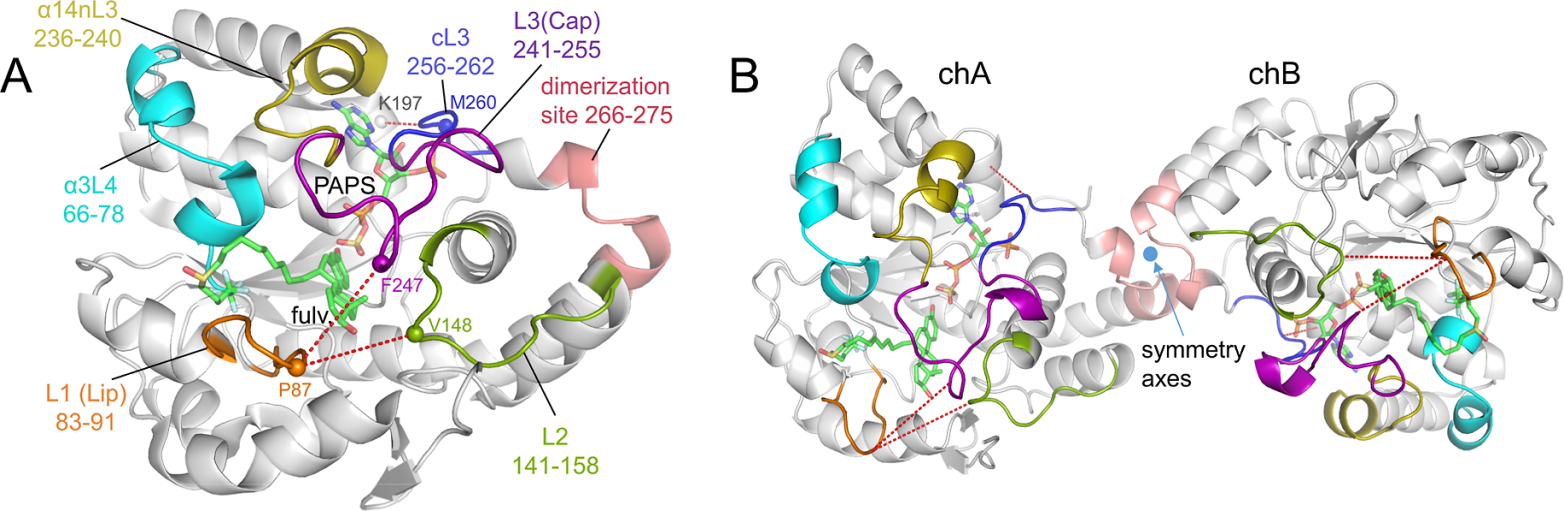
Structure of SULT1A1. (A) The monomer. Structural elements are presented: L1 (Lip) in orange, L2 in green, L3 (Cap) in magenta, cL3 in blue, α3L4 in cyan, α14nL3′ in gold, and the dimerization region in salmon. Residues used for distance calculations are labeled and represented as spheres. Fulvestrant and PAPS are depicted as sticks. (B) The dimer—composed of monomer chain A (chA) and chain B (chB).

The ligand-binding kinetic properties of SULTs have been studied over the years, with a consensus emerging that cytosolic SULTs follow a random order^11–14^ ligand binding, which results in the formation of a ternary complex between the protein, the cofactor PAPS, and the ligand.

Most cytosolic SULTs form dimers via a conserved dimerization site consisting of 10 residues with the consensus sequence KxxxTVxxxE (KTVE motif), near the C-terminus of the protein.^15^ Figure 1B represents the 3D structure of the SULT1A1 homodimer (PDB entry 2D06) (SULT1A1*2) via the KTVE dimerization C-terminal motif (AA 265–274 in 2D06). For SULT1A1*1, a crystal structure has been proposed with a homodimer formed by AA 217–225 helical interfaces (PDB entry 4GRA). However, this is an exception among reported SULT dimer structures and may represent a crystal structure artifact^16^ and will be not taken into account in this study. The biological significance of dimerization had previously been questioned since it has been reported that SULT1A1 monomers retain their activity in vitro.^10,17^ On the other hand, dimers show higher catalytic efficiency and stability compared to the monomeric form of the enzyme.^16^

Finally, dimerization in vivo through homodimers, or even heterodimers, may serve to stabilize the protein in the cell. The C-terminal dimerization motif is also essential for SULT interactions with other sulfotransferases like SULT4A1; mutating this motif indeed prevents dimer formation and disrupts functional interactions, with SULT4A1 able to regulate SULT1A1/3 levels in neuronal cells.^18^

In the work of Lu et al.,^17^ no significant impact of the dimerization on enzyme activity regarding a simple/small substrate was reported, and it was proposed that it only serves as a stabilizing factor, enabling the enzyme to withstand higher temperatures and urea concentrations without unfolding. In contrast, several studies on different SULT isoenzymes—SULT2A1, SULT1B1,^16^ SULT1E1,^19^ and SULT1A1 ^20^—suggested “half-site reactivity”. This “half-site reactivity” mechanism is the result of a global asymmetric structure in which each subunit of a 2-fold symmetric dimer likely isomerizes between two distinct states, each with a distinct capacity to interact with substrates or catalyze the given reaction.^10^

While this asymmetry is not apparent in crystal structures, the findings of half-reactivity reinforce the need for additional studies to gain insight into the catalytic mechanism of the SULT dimeric enzymes. Moreover, as the dimer interface is directly adjacent to the PAPS binding domain and active site cap of each SULT, such an interaction between the two subunits may play a key role in enzyme activity.

In order to elucidate the effect of dimerization on the dynamics and activity of SULT1A1, we performed comparative molecular dynamics (MD) simulations to measure the effects of cofactor and substrate binding on the monomeric and dimeric states of SULT1A1. A large ligand, fulvestrant, a 7α-alkylsulfinyl analogue of 17β-estradiol, was selected as the substrate. This selective estrogen receptor antagonist is used in the treatment of locally advanced or metastatic breast cancer in postmenopausal women. By using fulvestrant, we were able to explore the large conformational accommodating capacity of SULT1A1, a property characterized in our earlier work,^9^ whereas smaller ligands are generally accommodated without inducing substantial conformational rearrangements. Furthermore, this choice is also in agreement with previous experimental observations,^13^ which demonstrated that, for smaller substrates, the binding characteristics are largely similar between the monomeric and dimeric forms of SULT1A1. Thus, simulations on apo, PAPS-bound, and PAPS and a substrate fulvestrant-bound monomer and dimer were performed. Our results gave new insights into the mechanism of substrate and PAPS binding into the dimer, showing the implication of allosteric effects regulating the conformational dynamics of the SULT1A1 dimer.

## 2 Materials and Methods

### 2.1 Preparation of Studied Systems

#### 2.1.1 Monomeric Systems

The following systems were prepared for simulations (all simulated systems are summarized in Table 1): (i) SULT1A1 + PAPS monomer—using 4GRA^21^ PDB entry as the starting structure. (ii) SULT1A1 apoenzyme monomer (in the absence of bound cofactor or substrate)—starting also from 4GRA with the cofactor PAP being removed. (iii) SULT1A1+PAPS + fulvestrant monomer—the starting structure was taken from our previous publication based on docking.^9^

**Table 1:** Studies Systems

enzyme	starting structure
SULT1A1	4GRA
SULT1A1 + PAPS	4GRA
SULT1A1 + PAPS + fulvestrant	4GRA + simulation + docking
2SULT1A1	2D06
2SULT1A1 + 1PAPS	2D06
2SULT1A1 + 2PAPS	2D06
2SULT1A1 + 2PAPS + 2fulvestrant	2D06 with SULT1A1 + PAPS + fulvestrant monomer
2SULT1A1 + 2PAPS + 1fulvestrant	2D06 with SULT1A1 + PAPS + fulvestrant monomer

The cofactor PAP present in the 4GRA structure was replaced by PAPS. The PAPS structure was taken of SULT1E1 (PDB ID: 1HY347) and superposed to PAP in 4GRA by overlapping their common heavy atoms.

#### 2.1.2 Preparation of the Dimeric Systems

The 2D06 structure, available as a dimer, adopts the canonical dimerization motif, but with the SULT1A1*2 variant, which has a deleterious effect on substrate binding.^22^ To obtain the wild-type SULT1A1*1 sequence, the dimer was thus rebuilt by H213R substitution.

The RMSD of heavy atoms between one chain of 4GRA (SULT1A1*1) and 2D06 is 0.38 Å, showing a high similarity. 2D06 also includes the inactive cofactor PAP and the substrate estradiol in both chains. The PAP molecules were replaced with PAPS, by overlapping their common heavy atoms, and estradiol was removed. The parameters for PAPS and fulvestrant were taken from our previous study.^9^ PROPKA^23^ was used to determine the protonation states of titratable residues, at pH 7.0.

The following dimers were built using the above-described protocol: (iv) 2SULT1A1 + 2PAPS, (v) 2SULT1A1 + 1PAPS (with the removal of PAPS from the B chain), and (vi) 2SULT1A1 + 0PAPS (with the removal of both cofactors).

### 2.2 SULT1A1 + PAPS + Fulvestrant Dimer

The previously constructed SULT1A1 + PAPS + fulvestrant monomer was used to build the dimer with the dimer interface corresponding to 2D06. The coordinates of the protein, PAPS, and fulvestrant were duplicated and superposed onto each chain of the dimer using the crystal structure as a scaffold for the alignment. The alignment was performed on the rigid core residues of the protein (list of residues in SI) using PyMOL,^24^ ensuring precise positioning of the dimerization site while allowing the more flexible parts of the protein to naturally orient themselves. The resulting dimers were solvated as in the systems described above. The following fulvestrant containing dimers were built: (vii) 2SULT1A1 + 2PAPS + 2fulvestrant, (viii) 2SULT1A1 + 2PAPS + 1fulvestrant (with the removal of 1 fulvestrant from chain B), and (ix) 2SULT1A1 + 1PAPS + 1fulvestrant (removing PAPS from both chain A and fulvestrant from chain B).

The above-described sequential order for cofactor and substrate binding was selected since, in our previous study,^9^ the use of an MDeNM approach showed that PAPS-bound SULT1A1 exhibits sufficient flexibility to adopt conformations accommodating large substrates such as fulvestrant, 4-hydroxytamoxifen, or raloxifene. This finding led us to hypothesize that PAPS is bound first, structuring the cap before fulvestrant binding.

### 2.3 Solvation, Energy Minimization

All studied systems were solvated using the same protocol: the online web tool CHARMM-GUI^25,26^ was used to generate a solvent box of TIP3 water molecules around the protein, whose boundaries were at least 12 Å beyond the most distal part of the protein to prevent self-interaction across the periodic boundaries. The net charge of the system was balanced with Na^+^ ions, and further Na^+^ and Cl^‑^ ions were added for a simulated concentration of 0.15 mol/L.

Energy minimization was conducted with a series of progressively decreasing harmonic restraints applied to the positions of the heavy atom, beginning with the steepest descent (SD), where the harmonic force constant decreased every 100 steps, with values of 50, 10, 1, and 0.1 kcal/mol/Å^2^.

Following this, the harmonic restraints were released, and three cycles of 250 steps of SD and adopted basis Newton–Raphson (ABNR) minimizations were performed, followed by a final cycle of 500 steps. CHARMM^27^ was used to perform minimization, utilizing the additive all-atom CHARMM force field C36m.^28^ The system was then heated and equilibrated at 300 K for 100 ps in an *NVT* ensemble, followed by a 5 ns NPT equilibration run at 1 atm pressure using NAMD^29^ and the same force field mentioned above. Langevin dynamics with a damping coefficient of 1 ps-1 was used for constant temperature control, while the Nose–Hoover method with a piston oscillation period of 50 fs; a piston oscillation decay time of 25 fs was used for constant pressure control. The integration time step was set to 1 fs for the equilibration and 2 fs for the production run. For the energy calculations, the dielectric constant was set to 1. Electrostatic interactions were calculated using the particle mesh Ewald (PME) method, with a grid spacing of 1 Å or less, having an order of 6. The real space summation was truncated at 12.0 Å, and the width of the Gaussian distribution was set to 0.34 Å-1. van der Waals interactions were reduced to zero using a “switch” truncation operating between 10.0 and 12.0 Å.

### 2.4 MD Simulations

All atom molecular dynamics simulations were performed for the constructed systems using NAMD^29^ with the CHARMM force field C36m.^28^ For each system (I–IX), three 1 μs-long simulations were carried out starting from the equilibrated structures. For each simulation, random initial velocities were assigned according to the Maxwell–Boltzmann distribution at 300 K. The simulations were saved every 20 ps, resulting in 50,000 conformations per MD run and a total of 150,000 conformations per system. The parameters for the 1 μs production runs were identical with those used in the previously described 5 ns NPT equilibration.

### 2.5 Force Distribution Analysis (FDA)

Stress propagation was analyzed using the Force Distribution Analysis (FDA) tool, as implemented in GROMACS-FDA,^30^ based on GROMACS (2020.1).^31^ Trajectories under different simulation conditions (with or without the cofactor or ligand) were examined to compare residue-level pairwise forces across systems.

For each system, a 2D mean force matrix *F* was constructed, where each element *F*_*ij*_ represents the mean force acting between residues *i* and *j* over the trajectory.

External perturbation responses (cofactor and/or ligand binding) were assessed by computing the absolute difference between the mean force matrices of different systems, Δ*F* = |*F*_A_ – *F*_B_|.

Perturbation pathways connecting different protein regions were identified by constructing a connectivity graph within Δ*F*. The maximum cutoff value for absolute mean forces was determined by gradually increasing the cutoff and removing edges below it until further removal disrupted the perturbation connectivity between protein regions. Our approach ensures that the chosen cutoff maximizes noise reduction while preserving connectivity, allowing for a robust identification of stress propagation pathways.

## 3 Results and Discussion

### 3.1 Effects of Dimerization

To understand the effects of dimerization on the dynamical behavior of SULT1A1, we compared the apo enzyme in the monomer and dimer forms. RMSD over the three parallel simulations for each system was calculated after superimposing the backbone heavy atoms of each monomeric subunit constituting the dimer separately onto the equilibrated conformation. RMSD has shown an increase and has become asymmetric upon dimerization changing from 1.37 ± 0.13 Å for the monomer to 1.66 ± 0.24 Å and 1.44 ± 0.21 Å for the two subunits of the dimer (presented in Supporting Information Figure S1A,B).

To localize the regions responsible for the RMSD differences, we calculated the residue-based Root Mean Square Fluctuation (RMSF) for the monomer and dimer (Figure 2). These results show that the asymmetric increase in fluctuation upon dimerization is mostly concentrated to the helix-loop region formed by helix α14 and nL3—we will denote this region by α14nL3 (resid 228–240), which is far from the dimerization site (see Figure 1) and cL3.

**Figure 2:**
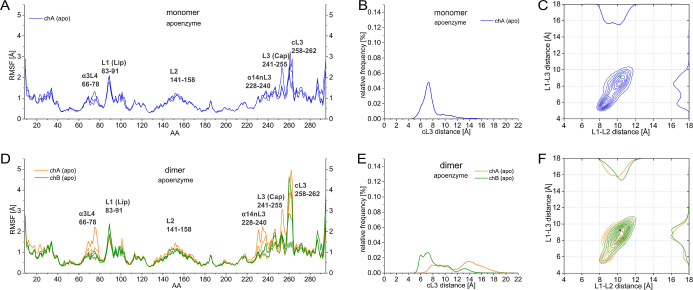
Effect of dimerization on the apoenzyme. RMSF of the Cα atoms per the amino acids (AA) in the MD simulations for the (A) monomer apoenzyme (blue); (D) of the dimer chains (orange/green). Distribution of cL3 distances in MD simulations for the (B) monomer apoenzyme (blue); (E) of the dimer chains (orange/green). Distribution of L1–L2 and L1–L3 distances for the (C) monomer apoenzyme (blue); (F) of the dimer chains (orange/green).

In order to analyze if the increased loop fluctuations can be directly associated with the opening of the gate at either the ligand binding or the PAPS binding site, we calculated distances that characterize the openness of these sites. To describe the openness of the ligand binding site gate, we used the distances L1-L2: defined between P87_Cα_ (tip of L1) and V148_Cα_ (tip of L2) and L1-L3: defined between P87_Cα_ (L1) and F247_Cα_ (tip of L3).^9^ Next, we characterized the openness of the cap over the nucleotide binding site (cL3) with the distance defined between the Cα of residues K197 (α15) and M260. These residues are denoted as spheres in Figure 1A.

By comparing the openness of cL3 for the monomer and dimer of the apo enzyme (Figure 2B,E), it is seen that subunit A of the dimer behaved significantly differently than the monomer, showing a widely spread population corresponding to more open conformations, ranging between 6 and 19Å compared to the population of the monomer being centered around 7 Å. Subunit B of the dimer showed a more modest opening: with increased population around 9 Å compared to the monomer, in general, mapping more open conformations than the monomer but less than subunit A. Thus, an asymmetricity of the dynamics of L3 in chA and chB opening can be seen.

For comparing the gate openness of the ligand binding site between the monomer and dimer, Figure 2C,F shows that the L1-L2 distance of the dimer’s subunit A exhibits similar behavior as the monomer, while the L1–L3 distance slightly opens up, with a diminished population corresponding to a 6 Å opening and a more populated 9 Å opening conformation compared to the monomer. Subunit B shows a more open conformation in both L1–L2 and L1–L3 distances compared to the monomer. The gate opening of the ligand binding site increased in both subunits upon dimerization showing asymmetric behavior, with a greater opening in subunit B. Interestingly, the monomer covers chA + chB as seen in Figure 2C,F, respectively. The 2D distribution graphs clearly show that while in the case of the monomer, the two-conformation-well map characterizing the ligand gate opening is similarly populated; in the case of the dimer, the population is shifted to the more open conformation showing a slight asymmetry between the two subunits of the dimer regarding L1–L2 opening. And we see that the whole big loop comprising nL3, L3, and cL3 opens up upon dimerization, particularly for chB. Moreover, our results also show an interesting asymmetry of the apo-dimer: while one subunit (A) exhibits more open cL3 conformation, the other subunit (B) shows a more open ligand binding gate conformation.

Supporting Information Figure S1C–F and Supporting Information Figures S2 and S3 show that there are no relevant differences of the RMSD, RMSF, and distances characterizing the loop openings between the PAPS-containing monomer and the 2PAPS-containing dimer and further between the PAPS + fulvestrant-containing monomer and the 2PAPS + 2fulvestrant-containing dimer (besides the asymmetricity of the cL3 opening). These results suggest that the binding of both cofactor and substrate has a stronger effect on the stabilization of the respective subunit than the dimerization effect in the presence of bound cofactor and ligand.

### 3.2 PAPS-Binding Effects on the Dimer Dynamics

In this section, we explore how cofactor (PAPS) binding alters the behavior of the dimer, both in terms of stabilizing individual subunits and inducing intersubunit effects.

For this reason, we compared the behavior of three different complexes: the apo dimer (without any ligands), a dimer with one PAPS molecule in subunit A (1PAPS dimer), and a dimer with PAPS bound to both subunits (2PAPS dimer).

The RMSD for subunit A of the dimer, containing the bound PAPS, decreased relevantly to 1.21 ± 0.15 Å compared to both subunits of the apo dimer (1.66 ± 0.24 Å and 1.44 ± 0.21 Å) (shown in Supporting Information Figure S4A,B). The PAPS-free B subunit maintained its RMSD value (1.47 ± 0.17 Å) similar to the subunits of the apo dimer. The 2PAPS dimer showed a decreased RMSD value in both subunits having values 1.23 ± 0.174 and 1.16 ± 0.16 Å, respectively (Supporting Information Figure S4C).

The RMSF values presented in Figure 3A,D,G show that in the 1PAPS dimer (Figure 3D), the PAPS-containing subunit A exhibits lower fluctuations at all functional loops and also stabilized the helix-loop region formed by residues 66–78 termed α3L4 and the α14nL3 helix-loop regions compared to both its PAPS-free subunit partner and the apo enzyme (Figure 3A). In the 2PAPS dimer (Figure 3G), the loops in both subunits are considerably more rigid, apart from cL3 that still shows relatively high fluctuation but with a lower magnitude compared to the PAPS-free containing subunits.

**Figure 3:**
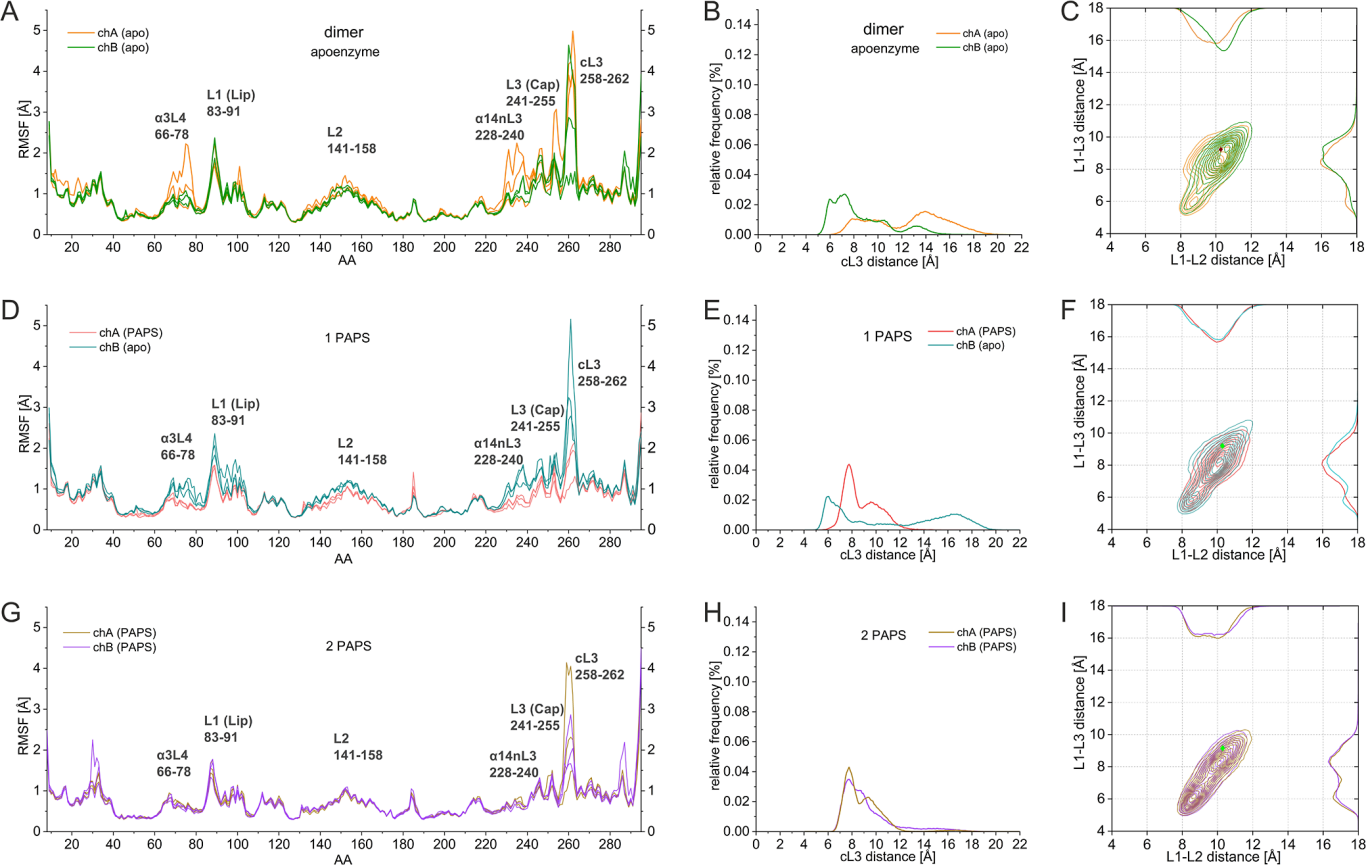
PAPS binding effect on dimers. RMSF of the Cα atoms per the amino acids (AA) in the MD simulations for the chains of (A) apodimer (orange/green); (D) 1PAPS dimer (salmon/teal); and (G) 2PAPS dimer (tan/violet). Distribution of cL3 distances in MD simulations for the chains of (B) apodimer (orange/green); (E) 1PAPS dimer (salmon/teal); and (H) 2PAPS dimer (tan/violet). Distribution of L1–L2 and L1–L3 distances for the chains of (C) apodimer (orange/green); (F) 1PAPS dimer (salmon/teal); and (I) 2PAPS dimer (tan/violet).

The opening of cL3 upon one-molecule PAPS binding can be seen in Figure 3B,E, indicating how PAPS binding to subunit A (chA) constrains the opening of cL3 from 6 to 19 Å to 6–12 Å. Interestingly, we observe that upon the binding of the first PAPS in chA, the opening of cL3 of chB without bound cofactor is increased from 5 to 15 Å to 5–19 Å, possibly facilitating the accommodation of the second PAPS in chB. In the same line, previously, Wang et al.^20^ proposed an energetic coupling between the active site caps of the adjacent subunit in the SULT1A1 dimer. The authors hypothesized that the first bound nucleotide may cause closure of the cap to which it is bound and, at the same time, stabilize the cap in the adjacent subunit in the open position, which corresponds to our observation for the PAPS-free subunit chB. Cap closure sterically may control active site access of the nucleotide and substrate; consequently, the structural changes in the cap occurring as a function of nucleotide occupancy can lead to changes in the substrate affinities and turnover of the enzyme.

In the 2PAPS dimer, where two PAPS molecules are bound in the two subunits, the cL3 opening is constrained (Figure 3H) in both subunits and similarly to the PAPS-bound subunit of the 1PAPS system (Figure 3H). The similarity of the openness of cL3 in the PAPS-containing subunit of the 1PAPS dimer and the two subunits of the 2PAPS dimer shows that the presence of the cofactor is the main factor for the rigidification of cL3. The effect of the dimerization could be noticed as an allosteric behavior: cL3 of the PAPS-free subunit B maps a wider conformational space compared to the apo enzyme upon PAPS binding in subunit A (Figure 3E compared to Figure 2B). Interestingly, it was previously discussed that the caps of SULT1A1 are controlled by homotropic allosteric interactions between PAPS molecules bound at the dimer’s active sites.^20^

Regarding the gate opening of the ligand binding site upon 1 PAPS binding, our findings suggest that upon the binding of the first PAPS, the gate of the same subunit slightly opens (L1–L2), and the PAPS-free subunit slightly closes (L1–L2 and L1–L3), exhibiting also a broader range of conformational distributions (Figure 3C,F). Further, our calculations showed that the fluctuations of chB are increased when 1 PAPS is bound to chA (Figure 3D), which is in concordance with facilitated large ligand binding in chB. Thus, we may speculate that the half-reactivity mechanism of the dimer is particularly important for large substrates.

Figure 3I shows that the gate opening is rigidified when two PAPS are bound at the same time, manifested on the one hand by the more restricted population distribution and on the other hand by the appearance of the L1–L2 closed conformations peaked around 9 Å and of L1–L3 at 6 Å. These data show that in the 2PAPS system, the gate opening of the ligand binding site rigidifies in a two-well conformation: being constrained in an open or closed conformation in the two subunits, restricting the conformational flexibility. These results are in line with experimental findings that at saturated PAPS concentrations, the substrate specificity of SULT1A1 is shifted toward smaller substrates.^21,32^

### 3.3 Fulvestrant Binding Effect on the PAPS-Containing Dimer

In this part, we examine how fulvestrant binding influences the dynamics of the 2PAPS-bound dimer, exploring the impact of progressive ligand occupancy on both local and distant structural elements. To follow this, we compared the behavior of the 2PAPS containing dimer with 2PAPS + 1fulvestrant (with fulvestrant in subunit A) and 2PAPS + 2fulvestrant-containing systems. By comparing the RMSD values of these systems, we observe that binding of 1 fulvestrant results in a considerable mobility increase of its own subunit (1.65 ± 0.37 Å), not changing the other subunit containing only PAPS (1.29 ± 0.17 Å, Supporting Information Figure S5A,B). However, upon binding of the second fulvestrant, the RMSD of the second subunit also increases (1.58 ± 0.37 and 1.70 ± 0.28 Å, respectively; Supporting Information Figure S5C).

The effects of fulvestrant binding on the RMSF values of the dimer are presented in Figure 4. Figure 4A,D shows that binding of one fulvestrant significantly increases the flexibility of L1, L2, α14nL3, L3, cL3, and α3L4 of the same subunit. With two fulvestrant monomers bound, all functional loops of both subunits of the dimer exhibit high flexibility (Figure 4G).

**Figure 4:**
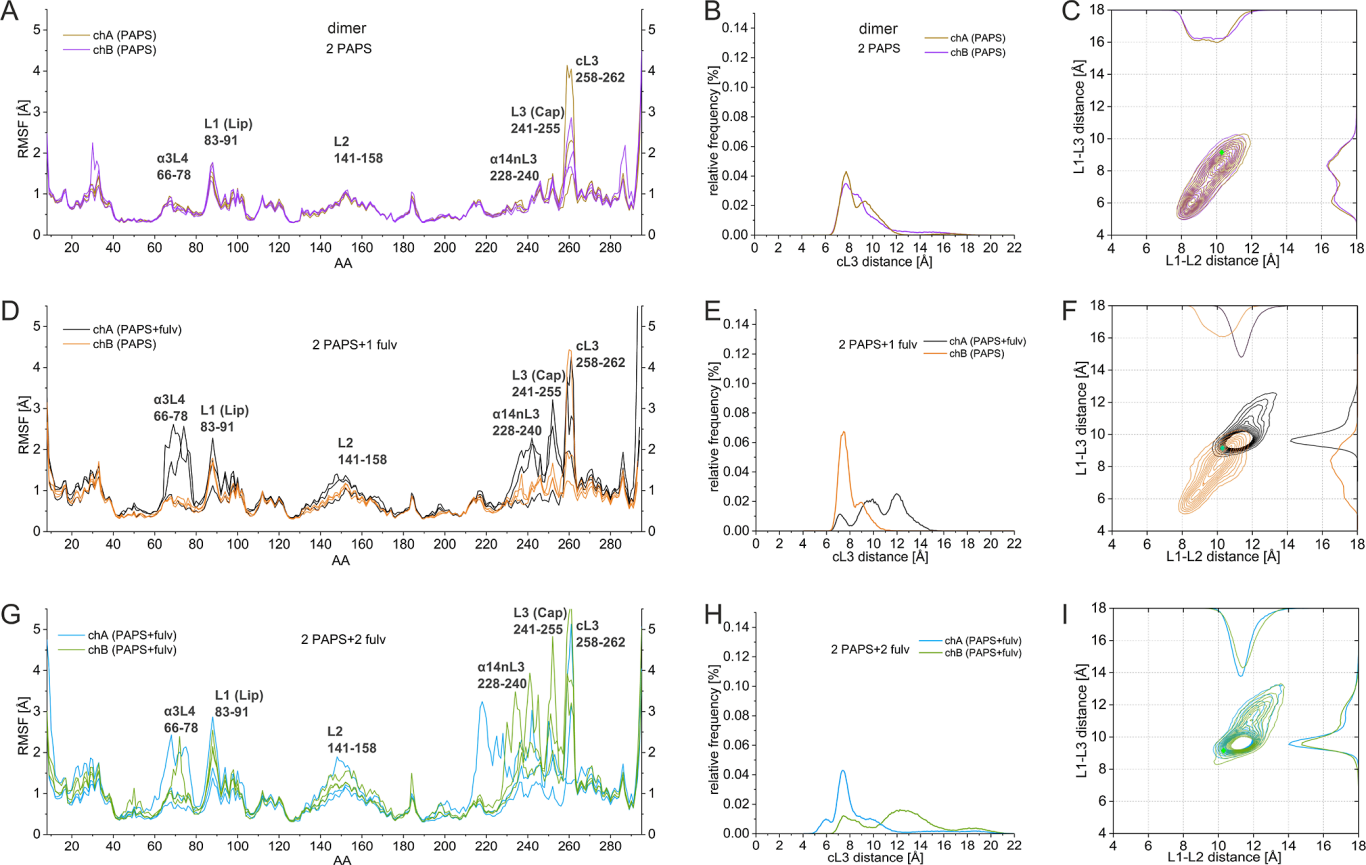
Fulvestrant binding effect on dimers. RMSF of the Cα atoms per the amino acids (AA) in the MD simulations for the chains of (A) 2PAPS dimer (tan/violet); (D) 2PAPS + 1fulv dimer (black/light brown); and (G) 2PAPS + 2fulv dimer (light blue/olive). Distribution of cL3 distances in MD simulations for the chains of (B) 2PAPS dimer (tan/violet); (E) 2PAPS + 1fulv dimer (black/light brown); and (H) 2PAPS + 2fulv dimer (light blue/olive). Distribution of L1–L2 and L1–L3 distances for the chains of (C) 2PAPS dimer (tan/violet); (F) 2PAPS + 1fulv dimer (black/light brown); and (I) 2PAPS + 2fulv dimer (light blue/olive).

For the extent of the cL3 loop openings upon fulvestrant binding, Figure 4B,E indicates that binding of one fulvestrant, besides maintaining the fulvestrant-less cL3 conformations with a reduced population, exhibits more opened conformations (the distribution being centered around 12 Å), in agreement with the increased cL3 fluctuations seen in Figure 4D. These results show that the binding of one fulvestrant affects not only the ligand-binding gate but also induces significant changes in cL3 of the same subunit shifting it toward the more open conformations.

Binding of the second fulvestrant results in an asymmetric cL3 opening, subunit A showing rather closed conformations (centered around 8 Å), while subunit B, besides displaying weekly populated closed conformations, exhibits widely open conformations centered around 13 Å as well (Figure 4H).

As discussed above, the gate opening of the ligand binding site for the 2PAPS systems (without a bound fulvestrant) shows a quite constrained conformational map for both subunits being restricted in two conformational pools (Figure 3C). Binding of one fulvestrant to subunit A shifts the conformational population restricting it to the open conformations (Figure 4F), in agreement with the increased L1 and L3 fluctuations. The gate opening of the fulvestrant-free subunit B retains it in rather closed conformations but explores a wider conformational space compared to the fulvestrant-free 2PAPS dimer, guessing the allosteric effects of the fulvestrant binding to subunit A.

Upon binding of the second fulvestrant, both gates behave similarly, showing the open, one-state population of the ligand binding gate, with the spread toward the open gate conformations exhibiting high degree of symmetry.

### 3.4 Allosteric Signaling within the Dimer

Given that homotropic allosteric interactions at the dimer’s active site can regulate SULT1A1 selectivity and catalytic efficiency, this section investigates the intra- and interdomain allosteric effects within the SULT dimer during cofactor and ligand binding.^20^

The results presented above reveal that PAPS binding, on the one hand, affects amino acids distant from the ligand binding pocket of its own subunit (decrease of RMSF of α3L4 and α14nL3 helix-loop regions) and, on the other hand, influences the behavior of the PAPS-free subunit of the dimer (cL3 and gate opening). To follow such allosteric effects in more detail, we performed FDA calculations on the apo- and the 1PAPS dimers. To follow the effect of the “perturbation” caused by PAPS binding, we first calculated the difference between the residue-based pairwise forces^30^ between the 1PAPS dimer and the apo dimer. Then, the residue-based punctual stress difference was calculated by summing up the absolute pairwise force differences sensed by a residue. These stress difference values were mapped onto the 3D structure of the dimer in a color-coded format, as shown in Figure 5, the maximal stress difference being denoted by red, the minimal by white.

**Figure 5:**
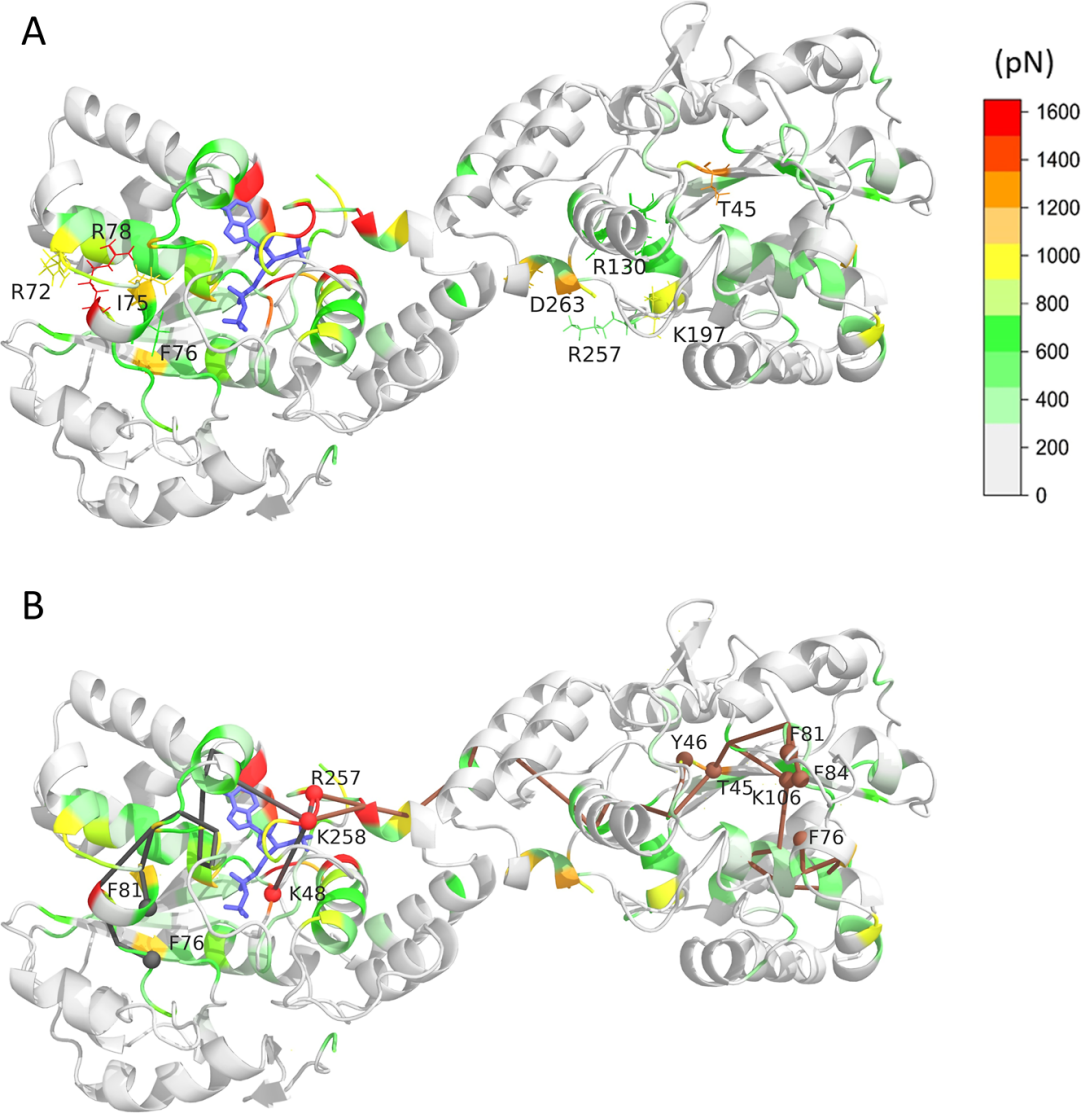
Punctual stress difference upon PAPS binding. (A) Color-coded representation of residue-level punctual stress difference between 1PAPS- and apo dimer, mapped on the 3D structure. PAPS is represented by blue sticks. Residues exhibiting significant stress alterations upon PAPS binding are labeled. (B) Pathways connecting residues exhibiting high punctual stress changes upon PAPS binding. Pathway origins and end points are indicated by red and gray/brown spheres, respectively. The intradomain pathway connecting the PAPS and fulvestrant binding residues is denoted by gray. The interdomain pathway linking the two PAPS and fulvestrant binding sites is colored brown.

Figure 5 shows that significant punctual stress differences are observed in both subunits of the dimer, showing a straightforward allosteric effect. Besides the high stress difference values of PAPS binding residues of subunit A (R257_A_, K258_A_, and K48_A_), relevant stress difference can be seen for amino acids remote from the PAPS binding pocket of the same subunit at residues of the α3L4 region: R72_A_, I75_A_, and R78_A_. In fact, this is the exact area where the RMSF shows a decrease in the fluctuation upon PAPS binding. Furthermore, increased stress difference can be observed at F142_A_, F76_A_, and F81_A_, which is part of the ligand binding pocket, and at K106_A_, which is a catalytically important residue. These results can be interpreted as the PAPS binding alters/prepares the ligand binding pocket for binding the ligand, showing an intrasubunit (i.e., within the PAPS bound subunit) allosteric effect upon PAPS binding.

Upon PAPS binding to subunit A, an intersubunit allosteric effect can also be detected by FDA calculations as well: the PAPS-free subunit B shows an increased punctual stress at the PAPS binding residues T45_B_, Y46_B_, and K106_B_; at R78_B_, R72_B_ of the α3L4 region; and at the ligand binding pocket, residues F81_B_, F84_B_, and F76_B_.

Figure 5B shows the path of the intra- and interdomain allostery, connecting the above-mentioned residues. Gray edges represent the path of the information transmitted from the PAPS binding residues (denoted by red spheres) to the ligand binding site of the same subunit crossing through α14nL3 and α3L4 secondary structure elements. The interdomain allosteric pathway is passing through K265_A_/E274_B_ of the dimerization site to the PAPS and ligand binding (path with brown edges) residues.

To understand the allosteric communication of the dimer upon fulvestrant binding, FDA calculations were executed by following the differences between the residue-based pairwise forces of the 2PAPS+1fulvestrant and the 2PAPS-containing dimer. The pairwise force differences were then summed to calculate the residue-based punctual stress difference, mapping them in a color-coded way onto the 3D structure of the dimer presented in Figure 6. Besides the observed increased punctual stress difference of residues F81_A_, F247_A_, F76_A_, and F84_A_ at the fulvestrant binging site of subunit A extending to residues of L3, α3, and α3L4, increased punctual stress difference can also be seen at the PAPS binding residues R257_A_, K48_A_, and S138_A_ of the same subunit. These are the same residues observed by FDA calculations upon PAPS binding discussed in the previous section.

**Figure 6:**
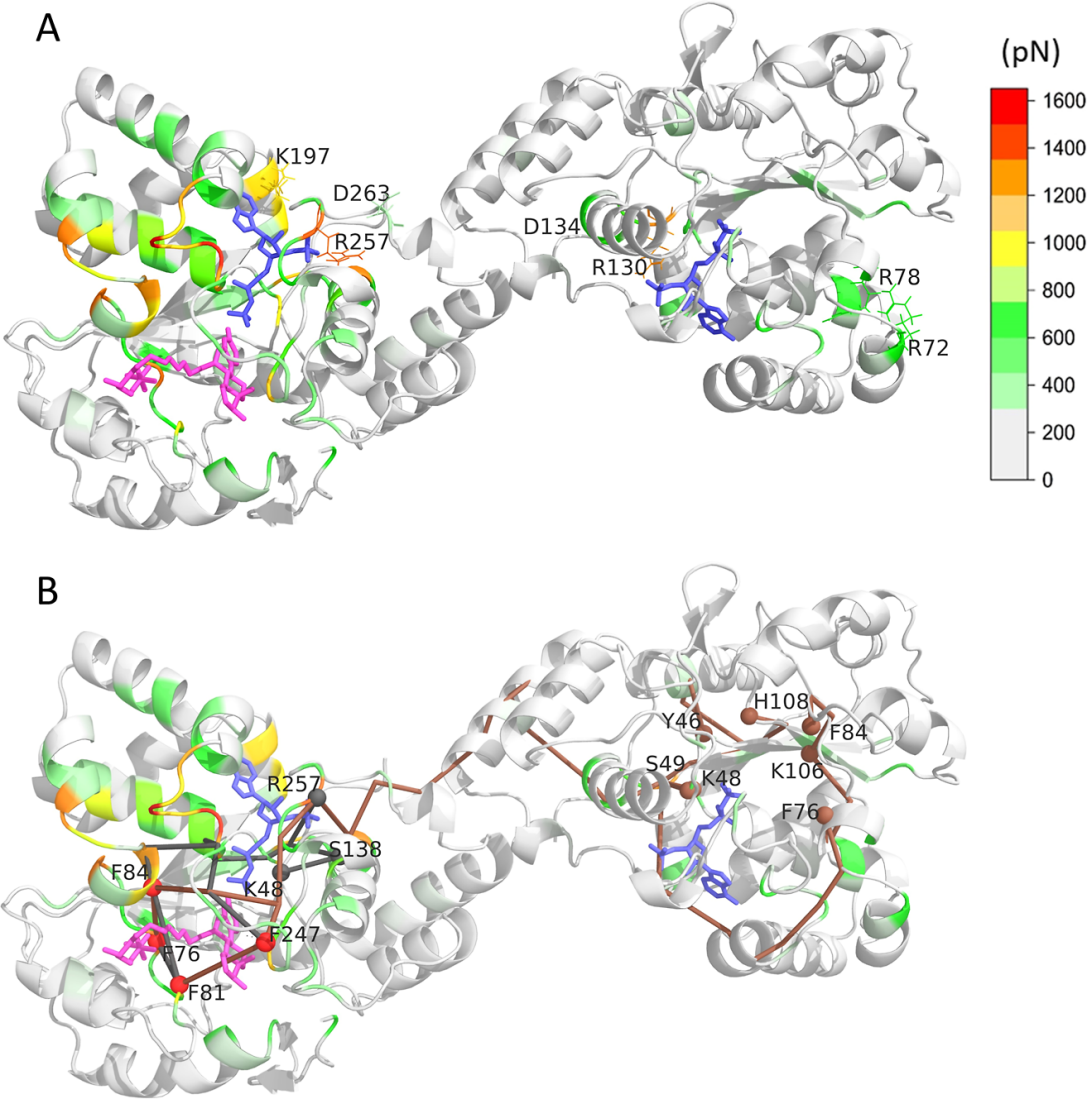
Punctual stress difference upon fulvestrant binding. (A) Color-coded representation of residue-level punctual stress difference between 2PAPS + 1fulvestrant and 2PAPS-containing dimer, mapped on the 3D structure. PAPS is represented by blue and fulvestrant by purple sticks. Residues exhibiting significant stress alterations upon PAPS binding are labeled. (B) Pathways connecting residues exhibiting high punctual stress changes upon fulvestrant binding. Pathway origins and end points are indicated by red and gray/brown spheres, respectively. The intradomain pathway connecting the PAPS and fulvestrant binding residues is denoted by gray. The interdomain pathway linking the two PAPS and fulvestrant binding sites is colored brown.

The data presented above support the existence of bidirectional allosteric communication between the cofactor-binding site and the ligand-binding site within a single subunit. Specifically, binding of PAPS is sensed at the ligand-binding site, and reciprocally, ligand binding is transduced to the PAPS-binding site.

Regarding intersubunit allostery, as previously discussed, the ligand binding gate of the subunit without fulvestrant of the 2PAPS + 1fulvestrant system explores a broader conformational space than the fulvestrant-free 2PAPS dimer. This is also being supported by the FDA calculations showing an increased punctual stress difference at the ligand binding residues K106_B_, H108_B_, F76_B_, and F84_B_ as well as at the PAPS binding residues K48_B_, S49_B_, and Y46_B_.

The pathway connecting the residues involved in intra- and interdomain allostery is presented in Figure 6B. Gray edges denote how the information is transmitted from the fulvestrant binding residues (denoted by red spheres) to the PAPS binding site of the same subunit through α14nL3 and α3L4 secondary structure elements, while the graph in brown shows how the information starting from the residues of the fulvestrant binding site passing through the PAPS binding site, crossing the dimerization surface at the same residue pair as the PAPS allosteric pathway K265_A_/E274_B_, reaches the ligand binding residues and at the end the PAPS binding residues in subunit B. Residues K48, R257, D263, K265­(A)/E274­(B), R275, D277, K133, D134, and R130 are the most robust, taking part of all four types of the above-mentioned pathways.

To further confirm that the observed allosteric effects originate specifically from enzyme dimerization, we performed FDA analysis on the monomeric form of the enzyme, as well. Supporting Information Figure S6A,B presents the effects of PAPS binding on the monomer and dimer, respectively. For the monomer, differences in residue-based pairwise forces were calculated between the PAPS-bound and the apo forms. For comparison, Supporting Information Figures S6 7B shows the corresponding punctual stress changes upon 1PAPS binding to the dimer. The magnitude of the PAPS-induced force changes in the monomer is substantially lower than in the PAPS-binding chain of the dimer with differences of approximately 200–400 pN at PAPS-binding residues. No significant perturbations were observed at fulvestrant-binding residues, indicating the complete absence of intradomain allosteric coupling in the monomeric state. Importantly, no significant perturbations were detected at fulvestrant-binding residues, confirming that intradomain allosteric coupling is entirely absent in the monomeric state upon PAPS binding.

A similar trend was observed for fulvestrant binding. Force differences between the 1PAPS + 1fulvestrant-bound monomer and the 1PAPS-bound monomer are shown in Supporting Information Figures S6C and compared with the corresponding differences in the 1fulvestrant-bound 2PAPS containing dimer (Supporting Information Figures S6D). Fulvestrant binding to the monomer produced again approximately 200–400 pN lower force changes at fulvestrant- and PAPS-binding residues, than in the fulvestrant-binding chain of the dimer. These minimal force changes further confirm that intradomain allosteric communication is greatly reduced in the monomer upon fulvestrant binding.

Given that intradomain allosteric effects are absent or markedly reduced in the monomer, and interdomain allosteric effects are inherently lacking, these results indicate that the allosteric behavior observed in the dimer arises from dimerization.

For SULTs functioning as a dimer, it was previously proposed that negative cooperativity between the PAPS binding sites of dimeric subunits is likely the driving force for half-site reactivity, and that the half-site reactivity could provide the system with directionality, favoring the release of products while promoting the binding of substrates.^10^ This hypothesis is strongly supported by our FDA results. Through FDA calculations for the first time, we described the allosteric communication between the active sites of the two subunits and how the information on PAPS-binding as well as substrate binding is relayed through the dimer complex.

## 4 Conclusions

Our study confirms that dimer formation plays a complex role in the mechanism of substrate binding and reactivity. The results show that in the case of the apo enzyme, fluctuation and the opening of functional loops increase upon dimerization, in this way enhancing cofactor and substrate recruitment. Furthermore, the two subunits of the apo dimer show an asymmetric flexibility, one being more flexible, mapping more open conformations than its counterpart and being more capable of binding the cofactor.

Upon binding of PAPS and fulvestrant to the dimer, our results show the presence of both intra- and interdomain allostery. The intradomain allostery is bidirectional: upon cofactor binding, the gate is influenced, and vice versa, ligand binding is transduced to the cap binding PAPS. For the interdomain allostery, we detected how upon binding of the PAPS/fulvestrant molecule to one subunit of the dimer both the PAPS and ligand-binding residues of the other subunit respond. We identified robust allosteric pathways connecting the two active sites for both PAPS and substrate binding and regions that the information is relayed through.

Interestingly, when two PAPS molecules are bound in the dimer, a high symmetry is observed, and the behavior is very similar to a SULT1A1 monomer, indicating that PAPS concentrations in the cells can modulate SULT activity behavior, as previously suggested.^33^

Altogether, these data allow us to speculate that the asymmetric behavior in the presence of one PAPS molecule in the dimer corresponds to the half-site reactivity discussed above, which may be particularly important for large substrates. Thus, our study brings further understanding of SULT1A1 structural dynamics and dimerization as related to enzyme function, with implications for large biologically relevant or pharmacological ligands.

## Supplementary Material



## Data Availability

The atomic coordinates of the starting structures are available via https://zenodo.org/records/15295187. The software and input parameters used in this study have been elaborated in the Computational Details section. The FDA is openly available at https://github.com/HITS-MBM/gromacs-fda, while NAMD is available at https://www.ks.uiuc.edu/Development/Download/download.cgi?PackageName=NAMD; charmm used for analysis in this work is available at https://academiccharmm.org/news/free-charmm.

## Notes

Dimerization effect—RMSD; dimerization effect on PAPS-bound systems; dimerization effect on PAPS + fulvestrant-bound systems; PAPS binding effect—RMSD; fulvestrant binding effect—RMSD; and punctual stress difference of the monomer and dimer upon PAPS and ligand binding (PDF)
