# Supplementary material for: Allosteric Mechanisms Triggering Substrate and Cofactor Binding in the SULT1A1 Dimer as Revealed by Molecular Dynamics Simulations
Source: J Chem Inf Model. 2025 Sep 30;65(19):10503–13. doi: 10.1021/acs.jcim.5c00845 (PMC12529757; doi:10.1021/acs.jcim.5c00845)
Supplement: Supplementary file 1 [file ci5c00845_si_001.pdf]

Supplementary Information for

"Allosteric mechanisms triggering substrate and cofactor binding in the SULT1A1 dimer as revealed by molecular dynamics simulations"

Daniel Toth[1,2], Balint Dudas[2,3], Arnaud B. Nicot[4], Maria A. Miteva[2]* and Erika Balog[1]*

[1]Department of Biophysics and Radiation Biology, Semmelweis University, 1094 Budapest, Hungary

[2] Université Paris Cité, CiTCoM UMR 8038 CNRS, INSERM U1268 MCTR, 75006 Paris, France

[3]Laboratory of Computational Biology, National Heart, Lung, and Blood Institute, National Institutes of Health, Bethesda, MD 20892, USA

[4]INSERM UMR 1064, Nantes Université, CR2TI, Nantes 44000, France.

Corresponding Author

Maria A. Miteva: maria.mitev@inserm.fr

Erika Balog: balog.erika@semmelweis.hu

Table of Contents

Contents **Page**

Figure S1. Dimerization effect – RMSD S3

Figure S2. Dimerization effect on PAPS- bound systems S4

Figure S3. Dimerization effect on PAPS+fulvestrant bound systems S5

Figure S4. PAPS binding effect – RMSD S6

Figure S5. Fulvestrant binding effect – RMSD S7

Figure S6. Punctual stress difference of the monomer and dimer upon PAPS and ligand binding. S8

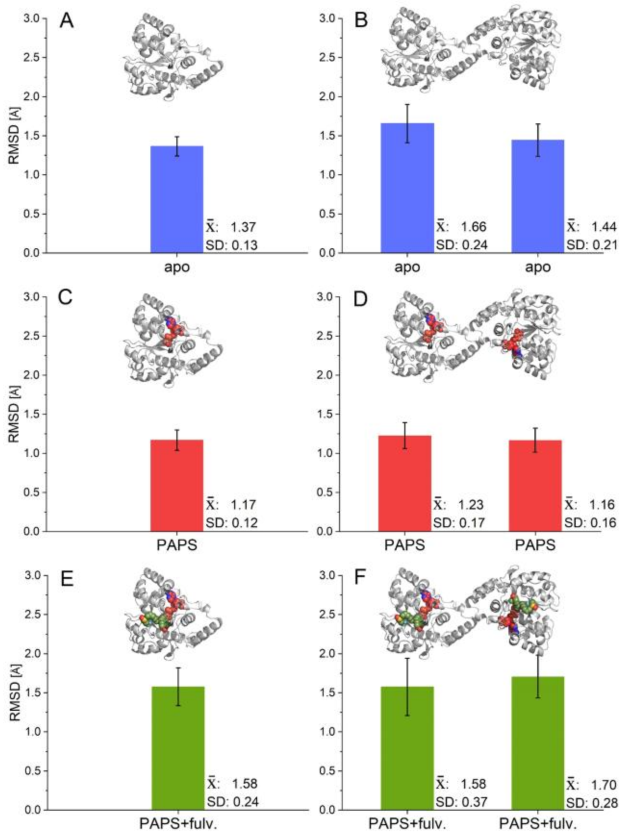

Figure S1. Dimerization effect – RMSDs calculated on the protein backbone, with respect to the crystal structure, averaged over the simulation time and parallel simulations. (**A/B**) represents the apoenzyme monomer/dimer form; (**C/D**) the PAPS-bound monomer/dimer; while (**E/F**) the PAPS+fulv bound monomer/dimer. Averages and standard deviations for each chain are represented in the bottom-right corner.

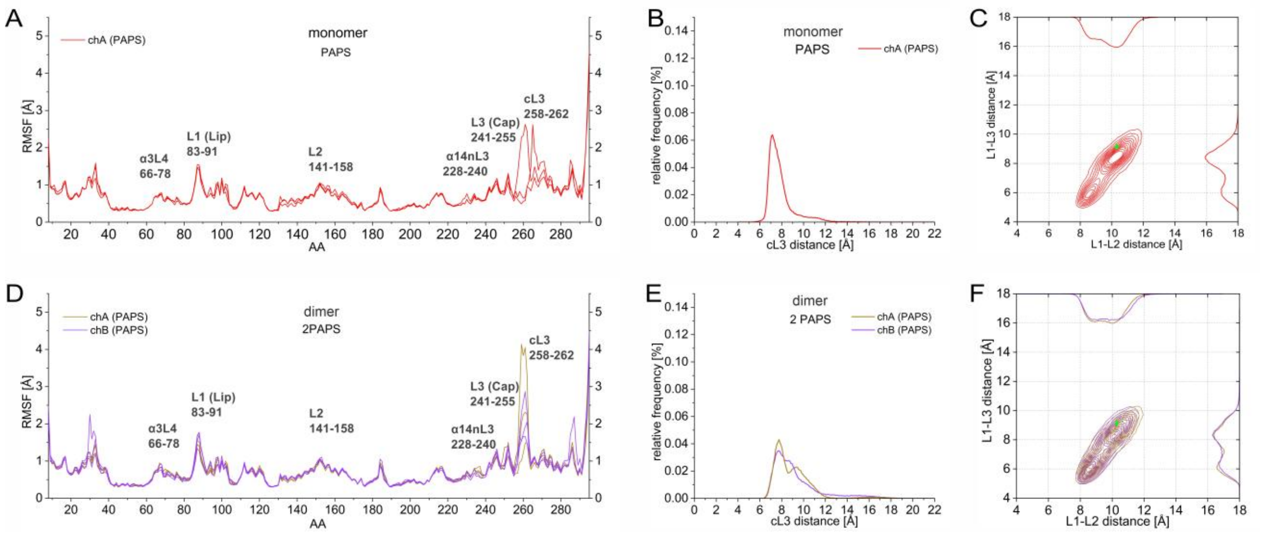

Figure S2. The effect of dimerization on PAPS bound enzyme. RMSF of the Cα atoms per the amino acids (AA) in the MD simulations for the (**A**) PAPS bound monomer (red); (**D**) 2PAPS dimer (tan/violet). Distribution of cL3 distances in MD simulations for the (**B**) PAPS bound monomer (red); (**E**) 2PAPS dimer (tan/violet). Distribution of L1-L2 and L1-L3 distances for the (**C**) PAPS bound monomer (red); (**F**) 2PAPS dimer (tan/violet).

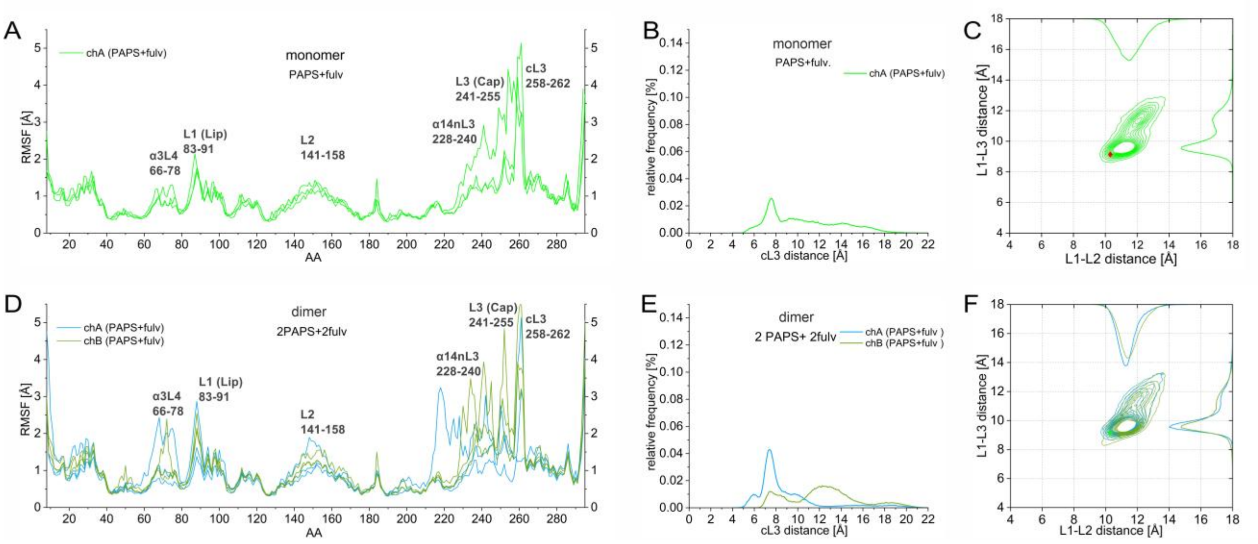

Figure S3. The effect of dimerization on PAPS+fulvestrant bound enzyme. RMSF of the Cα atoms per the amino acids (AA) in the MD simulations for the (**A**) PAPS+fulv bound monomer (lime); (**D**) 2PAPS+2fulv dimer (light blue/olive). Distribution of cL3 distances in MD simulations for the (**B**) PAPS+fulv bound monomer (lime); (**E**) 2PAPS+2fulv dimer (light blue/olive). Distribution of L1-L2 and L1-L3 distances for the (**C**) PAPS+fulv bound monomer (lime); (**F**) 2PAPS+2fulv dimer (light blue/olive).

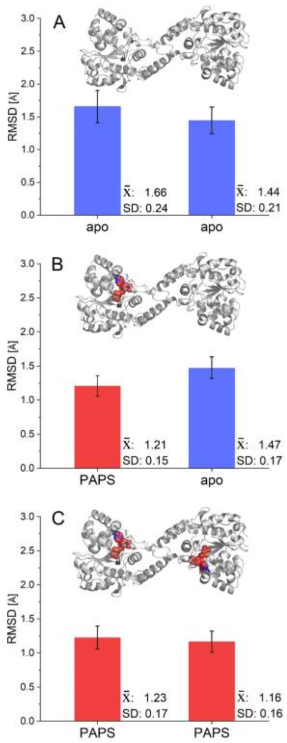

Figure S4. PAPS binding effect – RMSDs calculated on the protein backbone, with respect to the crystal structure, averaged over the simulation time and parallel simulations. (**A**) represents the apoenzyme dimer; (**B**) the 1PAPS dimer; while (**C**) the 2PAPS dimer. Averages and standard deviations for each chain are represented in the bottom-right corner.

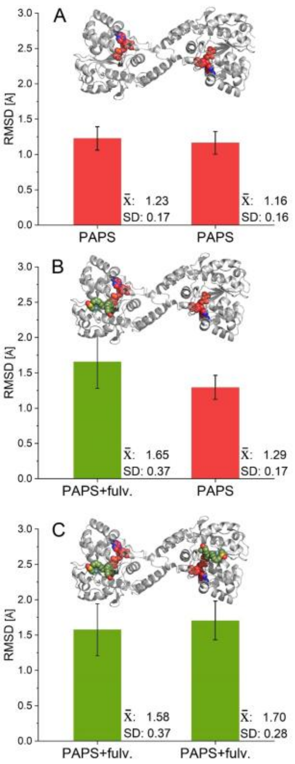

Figure S5. Fulvestrant binding effect – RMSDs calculated on the protein backbone, with respect to the crystal structure, averaged over the simulation time and parallel simulations. (**A**) represents the 2PAPS dimer; (**B**) the 2PAPS+1fulv dimer; while (**C**) the 2PAPS+2fulv dimer. Averages and standard deviations for each chain are represented in the bottom-right corner.

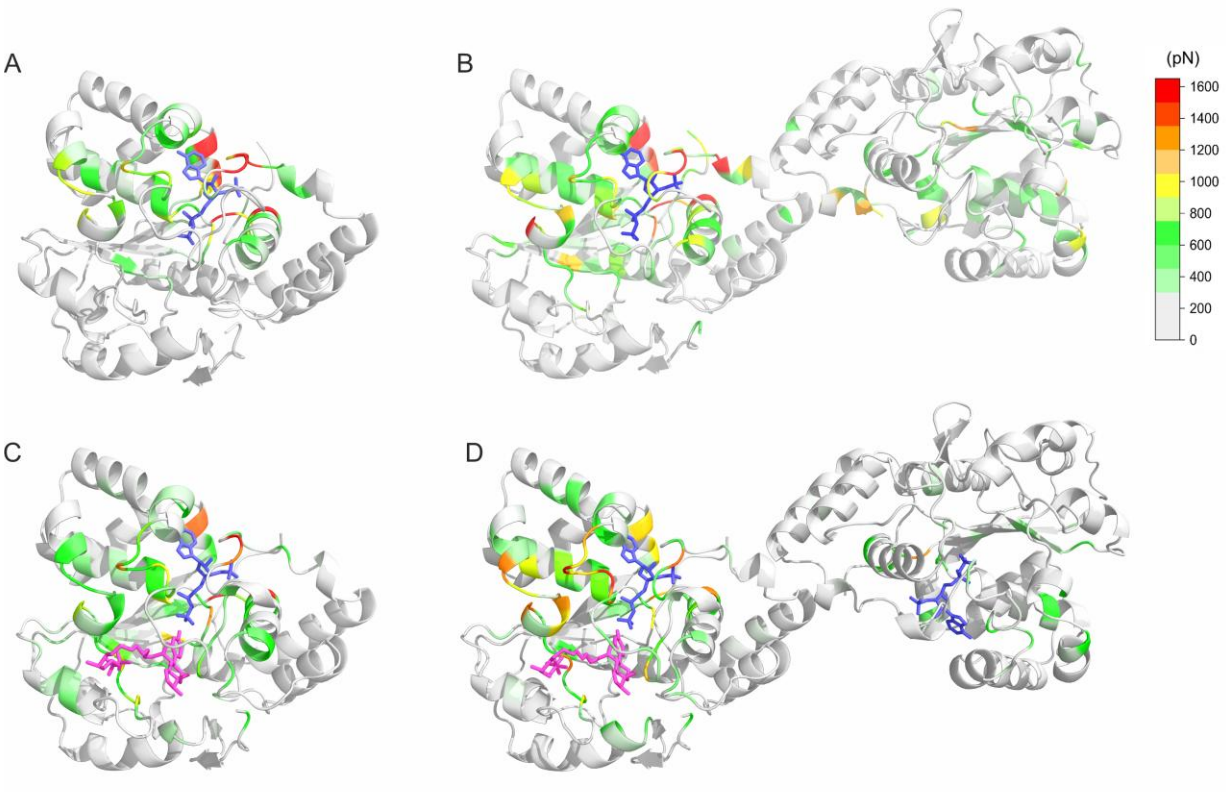

Figure S6. Punctual stress difference of the monomer and dimer upon PAPS and ligand binding. Color coded representation of residue-level punctual stress difference between (A) 1PAPS- and apo monomer, (B) 1PAPS- and apo dimer, (C) 1PAPS+1fulvestrant and 1PAPS containing monomer, (D) 2PAPS+1fulvestrant and 2PAPS containing dimer mapped on the 3D structure. PAPS is represented by blue-, fulvestrant by purple sticks.
